# *Borrelia burgdorferi*-mediated induction of miR146a-5p fine tunes the inflammatory response in human dermal fibroblasts

**DOI:** 10.1371/journal.pone.0286959

**Published:** 2023-06-15

**Authors:** Berta Victoria, Sarah A. Noureddine, Michael G. Shehat, Travis J. Jewett, Mollie W. Jewett

**Affiliations:** Division of Immunity and Pathogenesis, Burnett School of Biomedical Sciences, University of Central Florida College of Medicine, Orlando, Florida, United States of America; University of Kentucky College of Medicine, UNITED STATES

## Abstract

Colonization of a localized area of human skin by *Borrelia burgdorferi* after a bite from an infected tick is the first step in the development of Lyme disease. The initial interaction between the pathogen and the human host cells is suggested to impact later outcomes of the infection. MicroRNAs (miRNAs) are well known to be important regulators of host inflammatory and immune responses. While miRNAs have been shown to play a role in the inflammatory response to *B*. *burgdorferi* at late stages of infection in the joints, the contributions of miRNAs to early *B*. *burgdorferi* infection have yet to be explored. To address this knowledge gap, we used the published host transcriptional responses to *B*. *burgdorferi* in erythema migrans skin lesions of early Lyme disease patients and a human dermal fibroblasts (HDFs)/*B*. *burgdorferi* co-culture model to predict putative upstream regulator miRNAs. This analysis predicted a role for miR146a-5p in both, *B*. *burgdorferi-*infected skin and -stimulated HDFs. miR146a-5p was confirmed to be significantly upregulated in HDF stimulated with *B*. *burgdorferi* for 24 hours compared to uninfected control cells. Furthermore, manipulation of miR146a-5p expression (overexpression or inhibition) altered the *B*. *burgdorferi* driven inflammatory profile of HDF cells. Our results suggest that miR146a-5p is an important upstream regulator of the transcriptional and immune early response to early *B*. *burgdorferi* infection.

## Introduction

Lyme disease, caused by the bacterial pathogen *Borrelia burgdorferi*, is a multistage inflammatory disease with an estimated incidence of nearly 400,000 cases per year in the United States [1, 2]. *B*. *burgdorferi* is transmitted by the bite of an infected *Ixodes scapularis* tick. Events and interactions in the skin site of infection strongly influence the ability of *B*. *burgdorferi* to mount a productive disseminated infection [3–8], suggesting that vector-host-*B*. *burgdorferi* interactions in the skin are critical aspects of infection that drive downstream events and pathologies of Lyme disease. Immune dysregulation is a hallmark of Lyme disease [9]. However, much remains unknown about the mechanisms of immune dysregulation during early *B*. *burgdorferi* infection in the skin and the consequences for *B*. *burgdorferi* dissemination and late-stage pathologies.

MicroRNAs (miRNAs) are key post-transcriptional regulators of biological processes that have the potential to modulate nearly 30% of the protein-coding genes in the human genome [10, 11]. miRNAs are small non-coding RNA molecules that bind specific mRNA targets inhibiting their translation or affecting their stability [12, 13]. miRNAs act in a complex system of redundancy and compensation where one mRNA transcript may be modulated by multiple miRNAs. On the other hand, a single miRNA may modulate the expression of hundreds of mRNA targets. By targeting multiple genes involved in related biological processes, a single miRNA can have a large regulatory impact [14, 15]. There is increasing evidence for the importance of host miRNAs in controlling inflammatory responses and immunity during bacterial infections [16]. Furthermore, miRNAs have been implicated as drivers of infectious disease pathogenesis and suggested as potential therapeutic targets [16]. Although miRNAs have been documented to be expressed in *B*. *burgdorferi* stimulated astrocytes as a model of Lyme neuroborreliosis [17] and to contribute to late Lyme disease manifestations such as Lyme carditis and arthritis [17–20], miRNA-mediated regulatory mechanisms remain unexplored during the early stages of *B*. *burgdorferi* infection in the skin. To address this, we took advantage of two recent studies profiling host transcriptome changes during early infection with *B*. *burgdorferi* and conducted a reverse-engineered miRNA target analysis [21] to identify host upstream miRNA regulators which potentially impact the early immune response to *B*. *burgdorferi* infection in the skin.

## Materials and methods

### Reverse miRNA target prediction analysis

Reverse-engineered miRNA target analysis, as described previously [21], was applied to the transcriptome of erythema migrans skin lesions from early Lyme disease patients [22] and the transcriptome of normal human dermal fibroblasts stimulated with *B*. *burgdorferi* sensu lato for 24 hours [23] to identify upstream host miRNA regulators with predicted impact on early *B*. *burgdorferi* infection in the skin. Briefly, a list of strongly experimentally validated miRNA-mRNA interactions was downloaded from miRTarbase using the multimiR R software and hypergeometric tests conducted to assess overrepresentation of validated miRNA targets among the differentially expressed (DE) genes reported in the two datasets [22, 23].

### Generation of interaction networks and Venn diagrams

Filtered lists of putative upstream miRNA regulators and their overrepresented DE gene targets (>1 interaction, p<0.05, FDR<0.1) were used to construct interaction networks using Cytoscape [24]. Lists of filtered miRNAs (>1 interaction, p<0.05, FDR<0.1) were used to generate Venn diagrams using BioVenn [25].

### Bacterial strains and growth

*Borrelia burgdorferi* clone B31 A3 [26] was cultured in liquid Barbour-Stoenner-Kelly (BSK) II medium supplemented with gelatin and 6% rabbit serum [27] to logarithmic phase. The spirochetes were washed twice with phosphate buffered saline, pH 7.4 (PBS) and resuspended in 1 ml of PBS and the final density was determined using a Petroff-Hausser counting chamber under dark field microscopy. *B*. *burgdorferi* cultures were verified by PCR to contain the expected plasmid content [28].

### Fibroblast culture and stimulation

Primary Dermal Fibroblast; Normal, Human, Adult (HDFa, PCS-201-012, ATCC) were grown in fibroblast basal medium (PCS-201-030, ATCC), supplemented with fibroblast growth kit-serum-free (PCS-201-040, ATCC) and 10% fetal bovine serum (FBS) to passage 3 and aliquots stored at -80°C. Prior to co-culture with bacteria, a cryovial of fibroblasts was thawed and seeded on a T75 flask and the medium was gradually reduced in supplementation as follows: the day after thawing, the medium was replaced with fibroblast basal medium supplemented with L-Glutamine: 7.5 mM, rh FGF basic: 5 ng/mL, rh Insulin: 5 μg/mL, Ascorbic acid: 50 μg/mL, and 5% FBS. After two days, the cells were seeded at 1.5 x 10^5^ cells per well (or at 0.8 x 10^5^ for transfection experiments) in fibroblast basal medium with 2% FBS, in a 12-well plate. The cells were consistently assayed at passage 5. The following day, one day before bacterial stimulation, the medium was replaced with fibroblast basal medium without serum (or 1% FBS for the transfection experiments). Fibroblasts were stimulated with *B*. *burgdorferi* at a ratio of 100 bacteria per fibroblast for 2, 6 or 24 hours.

### RNA extraction

After removal of the supernatant for ELISA, total RNA extraction was performed using miRNeasy Tissue/Cells Advanced Mini Kit (217604, Qiagen). For the kinetic experiments, samples were harvested and frozen in QIAzol (79306, Qiagen) until completion of the experiment. Then, the samples were thawed, and total RNA extraction was completed using RNA isolation kit miRNeasy Mini Kit (217004, Qiagen) according to the manufacturer’s protocol, followed by treatment with DNase (79254, Qiagen).

### mRNA reverse transcription and qPCR

Between 0.5–1 μg of total RNA was reverse-transcribed using the iScript™ Select cDNA Synthesis Kit (1708897, BioRad), setting the reaction with the random primers mix and following the manufacturer’s instructions. Reverse transcriptase quantitative PCR (RT-qPCR) was performed using iQ SYBR Green supermix (1708882, BioRad) and the BioRad CFX 96 system. Primer sets used are listed in the S1 Table. Relative expression levels of the target transcripts were calculated using the 2^-ΔΔCt^ method [29]. *RPL2* was used as the endogenous control gene.

### miRNA reverse transcription and qPCR

Ten ng of total RNA was reverse-transcribed using TaqMan™ Advanced miRNA cDNA Synthesis Kit (A28007, Applied Biosystems), according to the manufacturer’s instructions. RT-qPCR was performed using TaqMan® Fast Advanced Master Mix (2X) (4444557, Applied Biosystems) and the TaqMan™ Advanced miRNA Assays (A25576, Applied Biosystems). The assays used are listed in the S2 Table. Relative expression levels of the studied miRNAs were calculated using the 2^-ΔΔCt^ method [29]. miR191-5p was used as the endogenous control.

### Transient transfection of miRNA mimics and inhibitors

mirVana miRNA mimics: hsa-miR-146a-5p (MC10722, Thermo Fisher Scientific) and Negative Control #1 (4464058, Thermo Fisher Scientific), mirVana® miRNA inhibitors: hsa-miR-146a-5p (ID MH10722) and Negative Control #1 (Ref 4464076), the fluorescence labeled oligo Silencer™ FAM-labeled Negative Control No. 1 siRNA (Ref. AM4620), and Opti-MEM® I Reduced-Serum Media, were purchased from Thermo Fisher Scientific, USA. The transfection reagent TransfeX™ ATCC® ACS-4005 was purchased from ATCC. The transient transfection of HDFa was optimized and assessed for efficiency. First, different amounts of oligo, transfection reagent volume and cell density, within the ranges recommended by the manufacture, were tested. Efficiency was assessed using a fluorescence labeled siRNA. Cell nuclei were also stained using Hoechst 33258 (Ref. H1399, Thermo Fisher Scientific, USA). Stained cells were observed and counted under a fluorescence microscope (Zeiss microscope, Zen 3.4 software). Second, the expression of miR146a-5p and some of its targets were assessed by RT-qPCR for transfection of miR146-5p mimic and the expression of some miR146a-5p targets was assessed for transfection of the miR146a-5p inhibitor. For the experiments, 50 nM of oligo (mimic or inhibitor) was used with 9 μl of transfection reagent per well, 24 hours before co-culture of HDFs with *B*. *burgdorferi*. Transfection of HDFs was performed according to manufacturer’s protocol for the oligos, mimics and inhibitors, and for the transfection reagent TransfeX™.

### Immunoblot blot analysis

After removing the media, HDFs were harvested using protein sample buffer for immunoblot analysis. Protein lysates were separated by SDS-PAGE and transferred to a PVDF membrane. Immunoblots were performed using rabbit anti-STAT1 (1:1000) (9172, Cell Signaling), rabbit anti-NF-ϰB p65 (1:1000) (D14E12, Cell Signaling) or rabbit anti-TRAF6 (1:500) (E2K9D, Cell Signaling) and mouse anti-actin (1:2000) (Clone C4, BD Biosciences) antibodies, in a 1:1 solution of Odyssey blocking buffer (LI-COR Biosciences) and Tris-buffered saline, pH 7.4 and 0.1% Tween20 (TBST) followed by IRDye 800CW goat anti-rabbit IgG and IRDye 680LT goat anti-mouse IgG (H+L) secondary antibodies (LI-COR Biosciences). Immunoblots were visualized and quantified using the LI-COR Odyssey scanner and software (Image Studio version 4). The fluorescence intensity for each protein target was normalized to the corresponding actin fluorescence intensity for each sample. The data are presented relative to a designated control sample for each data set, as indicated in the figure legends.

### IL6 ELISA

Interleukin 6 (IL6) secretion levels were measured in culture supernatants of HDF cells, by ELISA using the IL6 Human Uncoated ELISA Kit (88-7066-88, Thermo Fisher Scientific) following the manufacturer’s protocol.

### Statistical analysis

Statistical analyses were performed using GraphPad Prism (version 9.3.1, GraphPad) and an unpaired two tailed T-test analysis or Ordinary one-way ANOVA followed by the Welch and Brown-Forsythe ANOVA test for multiple comparisons.

The comparison of mRNA and miRNA expression levels between the groups was performed based on linear transformed data from the 2^-ΔΔCt^ results. For all statistical analyses, we considered the level of significance to be 5% (*P* < 0.05).

## Results

### Reverse-engineered miRNA target analysis predicted a role for miR146a-5p in the modulation of early transcriptional changes in response to *B*. *burgdorferi* infection

**Fig 1:**
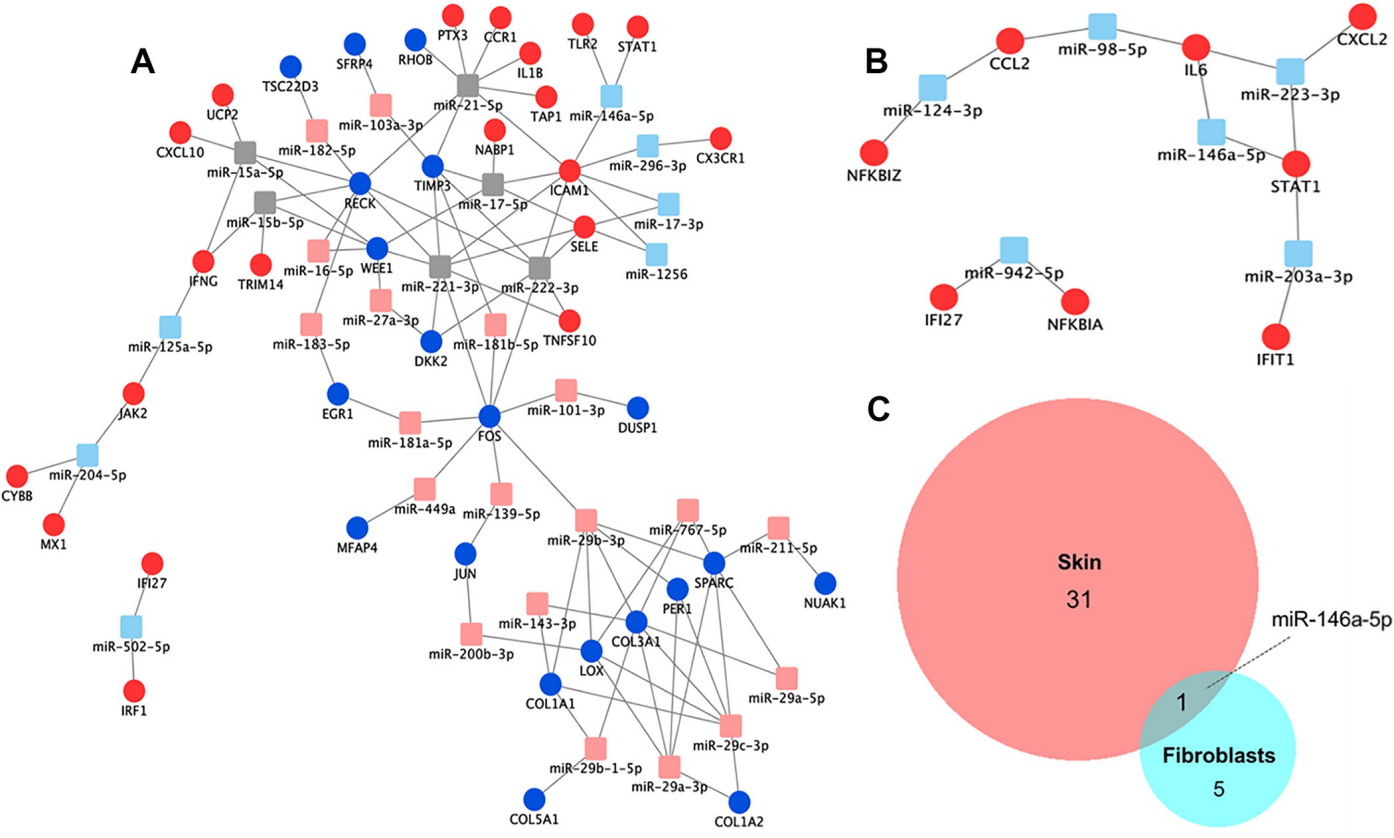
Upstream miRNA regulators predicted to influence the inflammatory response to early *B*. *burgdorferi* infection. The lists of predicted miRNAs and the overrepresented DE gene targets were filtered; >1 interaction, P<0.05, FDR<10%. (A) Upstream miRNA regulation of differentially expressed (DE) genes in erythema migrans skin lesions of early Lyme disease patients. DE genes (circles) reported by Marques *et al*. [22] and predicted upstream miRNA regulators (squares) that had an overrepresented number of DE gene targets. Red and blue circles represent the reported upregulated and downregulated genes, respectively. Light red and light blue squares represent predicted upregulated and downregulated miRNAs, respectively. Grey squares represent miRNAs that have an overrepresented number of gene targets in both the upregulated and downregulated sets of DE genes. (B) Upstream miRNA regulation of DE genes from primary human dermal fibroblasts (HDFs) in response to stimulation with *B*. *burgdorferi* sensu lato for 24 hours. Network representing strong validated interactions for miRNAs that had an overrepresented number of targets within the upregulated genes (red circles) reported by Meddeb *et al*. [23]. Light blue squares represent presumably down-regulated miRNAs. (C) Venn Diagram outlines common and unique predicted upstream miRNA regulators for the two sets of DE genes reported for skin lesions of early Lyme disease patients (skin) and *B*. *burgdorferi* stimulated HDFs (fibroblasts).

To identify potential miRNA regulators which may contribute to the host response to early *B*. *burgdorferi* infection we applied a reverse-engineered miRNA target analysis approach [21] to two published data sets which defined differentially expressed (DE) host genes in erythema migrans skin lesions collected from early Lyme disease patients [22] or human dermal fibroblasts (HDFs) stimulated with *B*. *burgdorferi* [23], as these are one of the cell subsets that *B*. *burgdorferi* is likely to encounter during early infection in the skin. This method was implemented by cross referencing the DE genes, both upregulated and downregulated, in each of the two published datasets [22, 23] with all known experimentally validated miRNA-mRNA interactions documented in the miRTarbase database [30], to obtain filtered lists of DE genes and the miRNAs that are validated to target them. The hypergeometric test was applied to each of these miRNAs to determine if transcripts confirmed to be targeted by these miRNAs were significantly enriched among the DE genes [22, 23]. To do this, for each miRNA, the proportion of target genes in the miRTarbase universe [30] (all possible mRNA targets) was compared to the proportion of target genes in the DE gene datasets (p< 0.05, FDR < 0.1). This analysis resulted in two prioritized lists of upstream miRNA regulators, one for each DE gene dataset, predicted to contribute to early transcriptional changes in response to *B*. *burgdorferi* infection. Finally, to maximize the potential impact of miRNA regulatory events, the prioritized lists of predicted upstream miRNA regulators and their DE gene targets were refined to include only those miRNAs interacting with more than 1 DE gene (>1 interaction, p<0.05, FDR<0.1). Predicted miRNA-mRNA interaction networks were modeled using Cytoscape [24] (Fig 1A and 1B). In total, this analysis of DE genes in the skin of early Lyme disease patients predicted 13 putative miRNA regulators based on upregulated genes and 25 putative miRNA regulators based on downregulated genes (Tables 1 and 2 and Fig 1A). Six of these miRNAs were predicted by both the upregulated and downregulated genes. Furthermore, this analysis of DE genes in HDFs in response to *B*. *burgdorferi* stimulation predicted 6 putative miRNA regulators based on the upregulated genes (Table 3 and Fig 1B). The analysis did not predict any putative miRNA regulators based on the downregulated genes in the *B*. *burgdorferi* stimulated HDF dataset. Strikingly, cross comparison of the miRNAs predicted from each of the datasets of DE genes revealed miR146a-5p was the single miRNA regulator predicted by both analyses (Fig 1C), suggesting a possible role of this miRNA regulator in control of the early host responses to *B*. *burgdorferi*.

**Table 1:** miRNA regulators predicted by reverse engineered miRNA targeting analysis of upregulated host genes in erythema migrans skin lesions collected from early Lyme disease patients.

	Based on Strongly Experimentally Validated miRNA-mRNA Interactions						
	Specific DE Study Set		Background (Universe of miRNA targets)				
Putative upstream miRNA regulator	Number of upregulated genes targeted by the specific miRNA	Total number of upregulated genes targeted by any miRNA	Number of genes targeted by the specific miRNA	Total number of genes targeted by any miRNA (Universe)	Fold Enrichment	P value	FDR^a^
hsa-miR-1256	2	46	3	2851	41.3	3.93E-06	8.26E-05
hsa-miR-296-3p	2	46	4	2851	31.0	1.56E-05	0.00026
hsa-miR-502-5p	2	46	5	2851	24.8	3.85E-05	0.00054
hsa-miR-17-3p	2	46	13	2851	9.5	0.00100	0.00603
hsa-miR-15a-5p^b^	3	46	45	2851	4.1	0.00545	0.01991
hsa-miR-222-3p^b^	3	46	45	2851	4.1	0.00545	0.01991
hsa-miR-204-5p	3	46	62	2851	3.0	0.01672	0.04683
hsa-miR-15b-5p^b^	2	46	35	2851	3.5	0.01794	0.04861
hsa-miR-21-5p^b^	5	46	137	2851	2.3	0.02117	0.05556
hsa-miR-221-3p^b^	3	46	72	2851	2.6	0.02745	0.06781
hsa-miR-146a-5p	3	46	74	2851	2.5	0.02999	0.06997
hsa-miR-17-5p^b^	3	46	84	2851	2.2	0.04477	0.09402
hsa-miR-125a-5p	2	46	51	2851	2.4	0.04776	0.09784

^a^False discovery rate

^b^Also predicted by downregulated genes

**Table 2:** miRNA regulators predicted by reverse engineered miRNA targeting analysis of downregulated host genes in erythema migrans skin lesions collected from early Lyme disease patients.

	Based on Strongly Experimentally Validated miRNA-mRNA Interactions						
	Specific DE Study Set		Background (Universe of miRNA targets)				
Putative upstream miRNA regulator	Number of downregulated genes targeted by the specific miRNA	Total number of downregulated genes targeted by any miRNA	Number of genes targeted by the specific miRNA	Total number of genes targeted by any miRNA (Universe)	Fold Enrichment	P value	FDR^a^
hsa-miR-29b-1-5p	3	24	10	2851	35.6	7.85E-07	2.15E-05
hsa-miR-767-5p	3	24	11	2851	32.4	1.23E-06	2.52E-05
hsa-miR-29a-5p	2	24	8	2851	29.7	2.86E-05	0.00029
hsa-miR-29c-3p	6	24	67	2851	10.6	7.27E-07	2.15E-05
hsa-miR-222-3p^b^	4	24	45	2851	10.6	2.65E-05	0.00029
hsa-miR-183-5p	2	24	25	2851	9.5	0.00107	0.00438
hsa-miR-139-5p	2	24	25	2851	9.5	0.00107	0.00438
hsa-miR-221-3p	5	24	72	2851	8.2	1.98E-05	0.00027
hsa-miR-103a-3p	2	24	29	2851	8.2	0.00166	0.00648
hsa-miR-29b-3p	6	24	93	2851	7.7	6.90E-06	0.00011
hsa-miR-449a	2	24	33	2851	7.2	0.00242	0.00828
hsa-miR-211-5p	2	24	34	2851	7.0	0.00264	0.00830
hsa-miR-15b-5p^b^	2	24	35	2851	6.8	0.00288	0.00830
hsa-miR-181b-5p	2	24	42	2851	5.7	0.00485	0.01173
hsa-miR-29a-3p	5	24	106	2851	5.6	0.00018	0.00139
hsa-miR-15a-5p^b^	2	24	45	2851	5.3	0.00590	0.01344
hsa-miR-143-3p	2	24	47	2851	5.1	0.00667	0.01478
hsa-miR-182-5p	2	24	52	2851	4.6	0.00884	0.01859
hsa-miR-101-3p	2	24	55	2851	4.3	0.01032	0.02030
hsa-miR-27a-3p	2	24	66	2851	3.6	0.01695	0.03021
hsa-miR-16-5p	2	24	67	2851	3.5	0.01764	0.03078
hsa-miR-181a-5p	2	24	74	2851	3.2	0.02297	0.03623
hsa-miR-200b-3p	2	24	77	2851	3.1	0.02550	0.03821
hsa-miR-17-5p^b^	2	24	84	2851	2.8	0.03196	0.04420
hsa-miR-21-5p^b^	3	24	137	2851	2.6	0.02563	0.03821

^a^False discovery rate

^b^Also predicted by upregulated genes

**Table 3:** miRNA regulators predicted by reverse engineered miRNA targeting analysis of upregulated host genes in human dermal fibroblasts stimulated with *B*. *burgdorferi*.

	Based on Strongly Experimentally Validated miRNA-mRNA Interactions						
	Specific DE Study Set		Background (Universe of miRNA targets)				
Putative upstream miRNA regulator	Number of upregulated genes targeted by the specific miRNA	Total number of upregulated genes targeted by any miRNA	Number of genes targeted by the specific miRNA	Total number of genes targeted by any miRNA (Universe)	Fold Enrichment	P value	FDR
hsa-miR-942-5p	2	19	6	2851	50.0	4.96E-06	5.06E-05
hsa-miR-223-3p	3	19	48	2851	9.4	2.28E-04	9.68E-04
hsa-miR-98-5p	2	19	34	2851	8.8	0.00132	0.00396
hsa-miR-203a-3p	2	19	60	2851	5.0	0.00676	0.01231
hsa-miR-146a-5p	2	19	74	2851	4.1	0.01207	0.01985
hsa-miR-124-3p	2	19	91	2851	3.3	0.02105	0.02752

^a^False discovery rate

### Validation of differentially expressed genes predicted to be enriched for miRNA targeting events during early *B*. *burgdorferi* infection and to be miR-146a-5p targets

**Fig 2:**
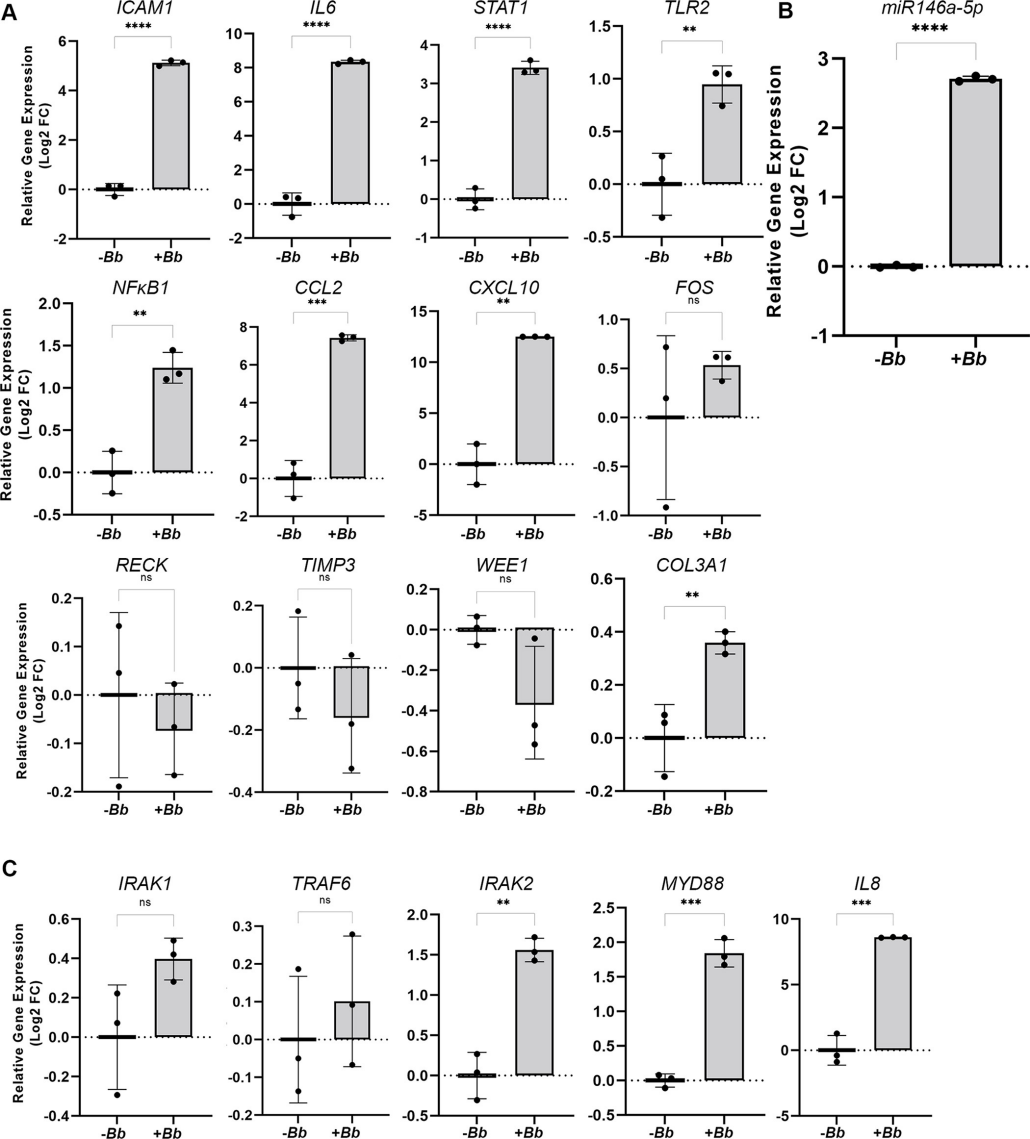
*B*. *burgdorferi* stimulation of HDFs resulted in increased expression of miR146a-5p and its targets of regulation. (A+C) RT-qPCR expression analysis of inflammatory genes and known targets of miR146a-5p regulation, normalized to *RPL2* gene expression. (B) RT-qPCR expression analysis of miR146a-5p, normalized to miR191-5p. Data are presented as the relative log fold change in mRNA or miRNA levels in HDFs co-incubated with (+*Bb*) or without (-*Bb*) *B*. *burgdorferi* for 24 hours. Data represent the average of biological triplicates ± standard deviation. Statistical significance was determined by unpaired t test (GraphPad, Prism). ns, not significant; **p < 0.01; ***p < 0.001; ****p < 0.0001.

The differentially expressed genes identified in the skin of early Lyme disease patients and HDF cells stimulated with *B*. *burgdorferi* both indicated transcriptional responses to *B*. *burgdorferi* characterized by strong induction of cytokine and IFN-associated genes [22, 23]. Similar findings were consistently reported in previous studies using fibroblast models of early *B*. *burgdorferi* infection [31–33]. In order to investigate the miRNA regulators predicted to contribute to the early response to *B*. *burgdorferi* infection, we applied an *in vitro* HDF cell/*B*. *burgdorferi* co-culture model. Using the mRNA-miRNA interactions predicted by our analysis, we first aimed to validate in this model the gene expression patterns of a panel of mRNA transcripts that were representative of predicted mRNA-miRNA interaction hubs (i.e., mRNAs with multiple predicted miRNA interactions): *COL3A1*, *ICAM1*, *FOS*, *RECK*, and *TIMP3* as well as those targeted by miR146a-5p: *IL6*, *STAT1*, and *TLR2* (Fig 1A and 1B). In addition, due to their known roles in proinflammatory signalling, the expression patterns of *NFϰB1*, *CCL2* and *CXCL10* were also examined [34]. Consistent with the transcriptional responses reported by Marques *et al*., 2017 and Meddeb *et al*., 2016 [22, 23], stimulation of HDFs with *B*. *burgdorferi* for 24 hours resulted in significant induction in the expression of *ICAM1*, *IL6*, *STAT1*, *TLR2*, *NFϰB1*, *CCL2*, and *CXCL10* (Fig 2A). In contrast, a subset of genes found to be downregulated in the skin during early *B*. *burgdorferi* infection [22], did not follow the same expression pattern in our model of HDFs exposed to *B*. *burgdorferi*. *FOS*, *RECK*, *TIMP3*, and *WEE1* showed no change in expression upon *B*. *burgdorferi* stimulation, while *COL3A1* was found to be upregulated in the presence of *B*. *burgdorferi* (Fig 2A), perhaps reflecting the multi-cellular, complex nature of human skin compared to dermal fibroblast cells alone. Together these data indicated that HDFs co-cultured with *B*. *burgdorferi* demonstrated a transcriptional profile consistent with signatures of inflammation.

The reverse-engineered miRNA target analysis predicted a number of putative miRNA regulators to be involved in the early response to *B*. *burgdorferi* (Fig 1 and Tables 1–3). miR146a-5p was predicted to be a negative regulator of *B*. *burgdorferi*-induced inflammation in early infection with mRNA targets including *TLR2*, *STAT1*, *ICAM1*, and *IL6* (Fig 1). Because these mRNA targets demonstrated increased expression in the presence of *B*. *burgdorferi* and miR146a-5p is a known negative regulator of these targets [30], in the most simplistic model one might predict decreased miR146a-5p expression in the presence of *B*. *burgdorferi*. Yet, miR146a-5p expression, similar to its mRNA targets (Fig 2A), was induced in HDFs stimulated with *B*. *burgdorferi* for 24 hours (Fig 2B). The expression levels of additional miRNAs predicted by the DE genes in the skin of early Lyme disease patients [22] and with evidence of expression in fibroblasts in the literature [35–37] were examined in our HDF model, but no changes in expression of these miRNAs were detected in the presence versus the absence of *B*. *burgdorferi* (S1 Fig).

Based on the current understanding of the inflammatory response pathways involving TLR2/NF-ϰB activation and modulation by miR146a-5p, we also assessed the expression of genes *IRAK1*, *TRAF6*, *IRAK2*, *MYD88*, and *IL8* to garner additional insight into the regulatory role of this miRNA in the early inflammatory response to *B*. *burgdorferi*. Gene expression of *IRAK1* and *TRAF6*, which are the most common miR146a-5p targets known to be inhibited in a negative feedback axis, was unchanged in HDF cells stimulated with *B*. *burgdorferi* for 24 hours (Fig 2C). However, IRAK2, another kinase that supports NF-ϰB activation and is targeted by miR146a-5p [38, 39], was upregulated when the cells were exposed to *B*. *burgdorferi* stimulation (Fig 2C). The adaptor molecule *MYD88* and *IL8* also showed a strong induction, as expected [22, 23], in response to *B*. *burgdorferi* (Fig 2C).

### The kinetics of miR146a-5p induction are consistent with a model of a NF-ϰB-dependent hierarchical regulatory network

**Fig 3:**
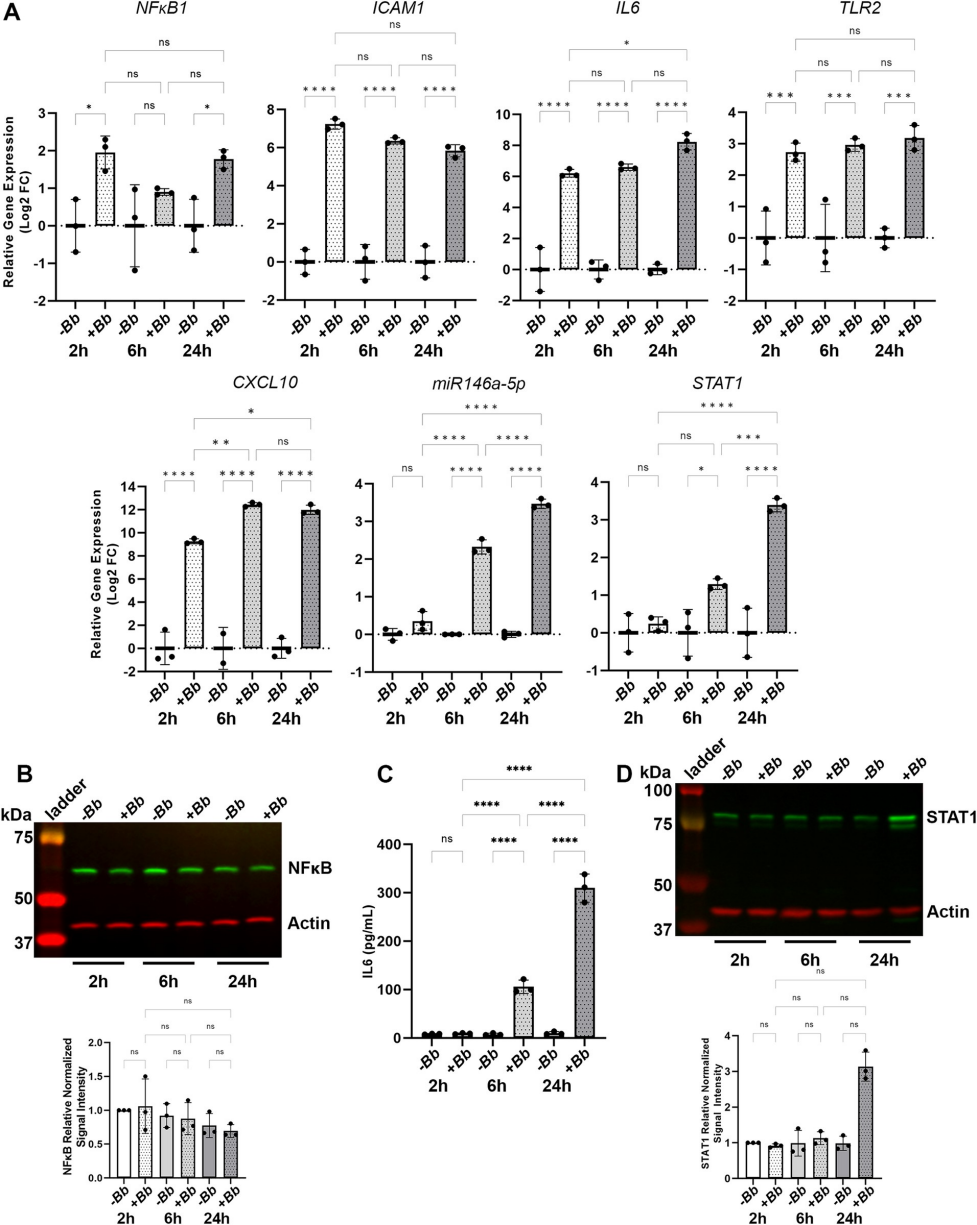
The kinetics of expression miR146a-5p and miR146a-5p-related genes in *B*. *burgdorferi* stimulated HDFs are consistent with a model of a NF-ϰB-dependent hierarchical regulatory network. (A) RT-qPCR expression analysis of inflammatory genes and miR146a-5p, normalized to *RPL2* and to miR-191-5p gene expression, respectively. RT-qPCR data are presented as the relative log fold change in mRNA or miRNA levels in HDFs co-incubated with *B*. *burgdorferi* (+*Bb*) for 2 hours, (2 h), 6 hours (6 h) or 24 hours (24 h) compared with HDFs alone (-*Bb*) at each time point. (B) Immunoblot analysis of NF-ϰB and actin levels produced by HDFs co-incubated with *B*. *burgdorferi* (+*Bb*) compared with HDFs alone (-*Bb*) at each time point. Molecular weights are shown in kilodaltons (kDa). Representative data of three biological replicates are shown. Quantification of the NF-ϰB protein levels in each sample normalized to actin and relative to the NF-ϰB/actin signal intensity of HDFs alone (-*Bb*) at 2 hours. Data represent the average of biological triplicates ± standard deviation. (C) Quantification of the picograms per milliliter (pg/mL) of IL6 secreted by HDFs co-incubated with *B*. *burgdorferi* (+*Bb*) compared with HDFs alone (-*Bb*) at each time point. Data represent the average of biological triplicates ± standard deviation. (D) Immunoblot analysis of STAT1 and actin levels produced by HDFs co-incubated with *B*. *burgdorferi* (+*Bb*) compared with HDFs alone (-*Bb*) at each time point. Molecular weights are shown in kilodaltons (kDa). Representative data of three biological replicates are shown. Quantification of the STAT1 protein levels in each sample normalized to actin and relative to the STAT1/actin signal intensity of HDFs alone (-*Bb*) at 2 hours. Statistical significance was determined by Ordinary one-way ANOVA followed by the Welch and Brown-Forsythe ANOVA test for multiple comparisons (GraphPad, Prism). ns, not significant; *p< 0.05; **p < 0.01; ***p < 0.001; ****p < 0.0001.

To understand the kinetics of *B*. *burgdorferi* induction of miR146a-5p and representative target genes, HDF cells were co-cultured with *B*. *burgdorferi* for 2, 6 and 24 hours and gene expression analysis was performed. *miR-146a-5p* expression has been shown to be controlled by NF-ϰB [40] and miR146a-5p is itself a negative regulator of NF-ϰB [41]. *NF*ϰ*B1* expression was found to be upregulated in HDF cells in the presence of *B*. *burgdorferi* at the 2-hour and 24-hour time points but was not found to be significantly induced at the 6-hour time point (Fig 3A). Consistent with the known pattern of gradual NF-ϰB-dependent upregulation [41], miR146a-5p expression showed no significant induction at the 2-hour time point but demonstrated a significant increase in expression at the 6-hour and 24-hour time points following *B*. *burgdorferi* stimulation (Fig 3A). NF-ϰB regulated genes, *ICAM1* [42, 43], *IL6* [44–46] and *TLR2* [47, 48], on the other hand, demonstrated significant induction at the earliest point of 2 h, which was maintained throughout the 24-hour time course (Fig 3A). *STAT1* expression is regulated by interferons [49], which themselves are regulated by NF-ϰB [50, 51]. A pattern of gradual upregulation of *STAT1* in *B*. *burgdorferi* stimulated cells, similar to that of miR146a-5p was observed (Fig 3A). Analysis of targets, NF-ϰB, IL6 and STAT1, was expanded to examine the kinetics of protein production in *B*. *burgdorferi* stimulated HDF cells by immunoblot or ELISA. Despite the observed *B*. *burgdorferi*-dependent increases in *NFϰB1* expression, no significant differences in NF-ϰB protein levels were detected at any of the time points (Fig 3B). A significant increase in IL6 protein levels was detected following 6 hours of stimulation, with even greater IL6 production measured at 24 hours (Fig 3C). STAT1 protein levels were unchanged at 2 hours and 6 hours, however at 24 hours a strong trend in increased protein production was detected in the stimulated cells (Fig 3D, *P* = 0.08, +*Bb* 2 hours vs. +*Bb* 24 hours). Together these data are consistent with a model of *B*. *burgdorferi* stimulation of HDF cells resulting in rapid early upregulation of expression of *NFϰB1* and some of the NF-ϰB-regulated genes, followed by upregulation of miR146a-5p and *STAT1*.

### Manipulation of miR146a-5p levels resulted in altered *B*. *burgdorferi*-mediated inflammatory gene expression by HDF cells

**Fig 4:**
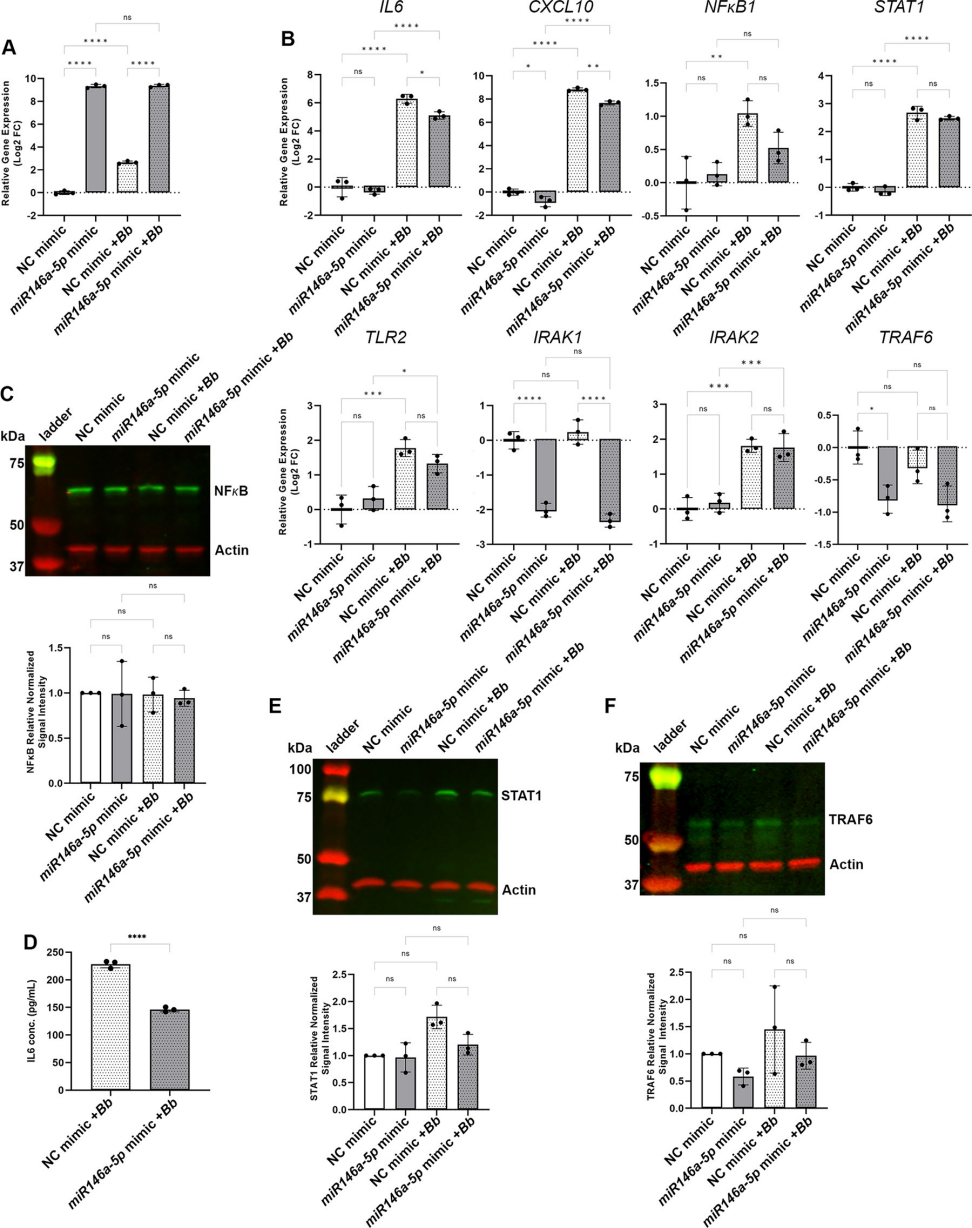
. Overexpression of miR146a-5p reduced the inflammatory response of *B*. *burgdorferi* stimulated HDFs. HDF cells were transfected with the miR146a-5p mimic or the negative control (NC) mimic and stimulated with (+*Bb*) or without *B*. *burgdorferi* for 24 hours. (A) RT-qPCR expression analysis of miR146a-5p, normalized to miR191-5p. (B) RT-qPCR expression analysis of miR146a-6p target and related genes, normalized to *RPL2* gene expression. RT-qPCR data are presented as the relative log fold change in miRNA or mRNA levels in cells transfected with the miR146a-5p mimic compared to cells transfected with the negative control mimic, in the absence or presence of *B*. *burgdorferi*. Data represent the average of biological triplicates ± standard deviation. Statistical significance was determined by Ordinary one-way ANOVA followed by the Welch and Brown-Forsythe ANOVA test for multiple comparisons (GraphPad, Prism). ns, not significant; *p< 0.05; **p < 0.01; ***p < 0.001; ****p < 0.0001. (C) Immunoblot analysis of NF-ϰB and actin levels produced by the HDFs. Molecular weights are shown in kilodaltons (kDa). Representative data of three biological replicates are shown. Quantification of the NF-ϰB protein levels in each sample normalized to actin and relative to the NF-ϰB/actin signal intensity of HDFs alone treated with the negative control mimic (NC mimic). Data represent the average of biological triplicates ± standard deviation. (D) Quantification of the picograms per milliliter (pg/mL) of IL6 secreted by the HDFs. Data represent the average of biological triplicates ± standard deviation. Statistical significance was determined by unpaired t test (GraphPad, Prism). ****p < 0.0001. (E) Immunoblot analysis of STAT1 and actin levels produced by the HDFs. Molecular weights are shown in kilodaltons (kDa). Representative data of three biological replicates are shown. Quantification of the STAT1 protein levels in each sample normalized to actin and relative to the STAT1/actin signal intensity of HDFs alone treated with the negative control mimic (NC mimic). Data represent the average of biological triplicates ± standard deviation. (F) Immunoblot analysis of TRAF6 and actin levels produced by the HDFs. Molecular weights are shown in kilodaltons (kDa). Representative data of three biological replicates are shown. Quantification of the TRAF6 protein levels in each sample normalized to actin and relative to the TRAF6/actin signal intensity of HDFs alone treated with the negative control mimic (NC mimic). Data represent the average of biological triplicates ± standard deviation.

**Fig 5:**
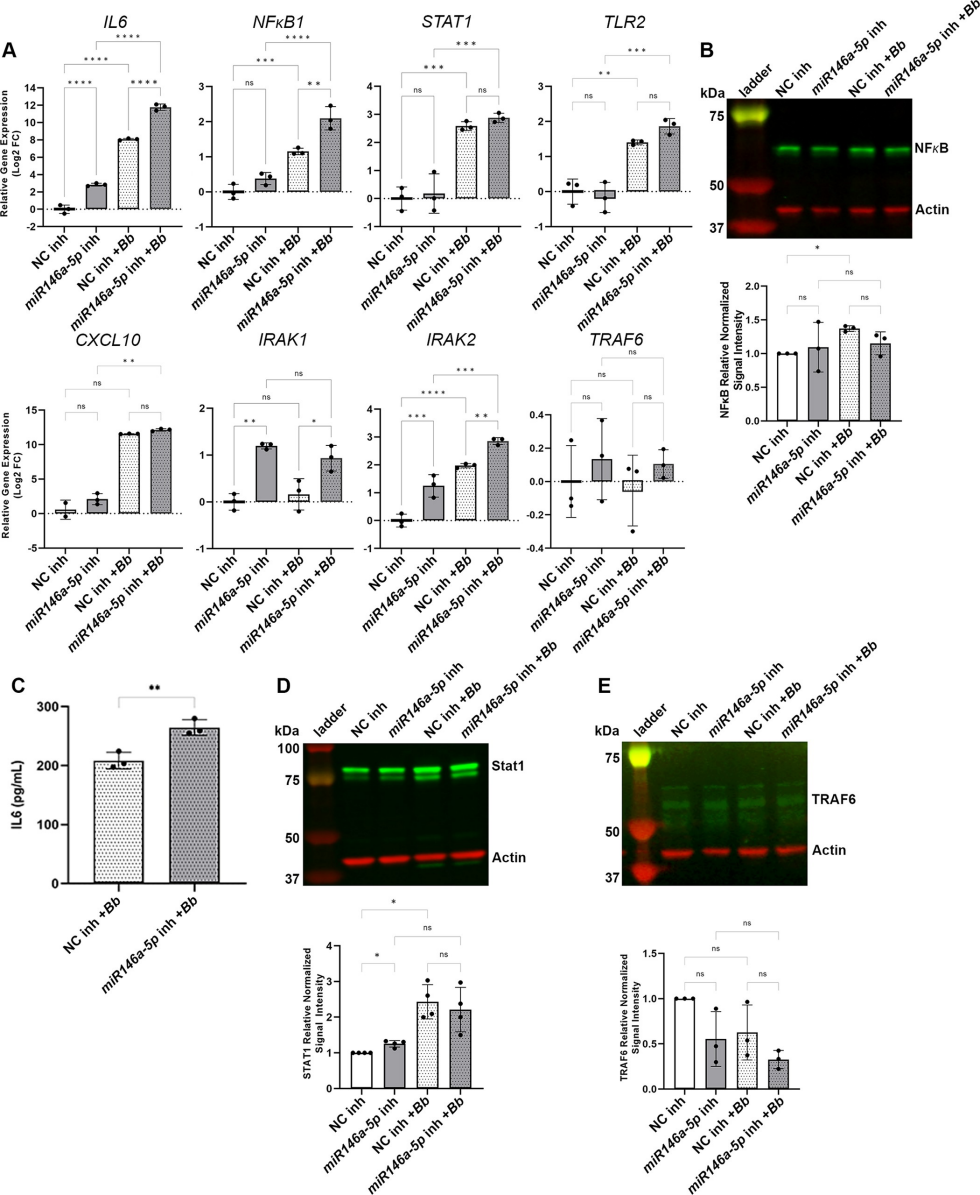
Inhibition of miR146a-5p resulted in increased expression of inflammation associated genes in *B*. *burgdorferi* stimulated HDFs. HDF cells were transfected with the miR146a-5p specific inhibitor (miR146a-5p inh) or the negative control inhibitor (NC inh) and stimulated with (+*Bb*) or without *B*. *burgdorferi* for 24 hours. (A) RT-qPCR expression analysis of miR146a-6p target and related genes, normalized to *RPL2* gene expression. RT-qPCR data are presented as the relative log fold change in mRNA levels in cells transfected with the miR146a-5p inhibitor compared to cells transfected with the negative control inhibitor, in the absence or presence of *B*. *burgdorferi*. Data represent the average of biological triplicates ± standard deviation. Statistical significance was determined by Ordinary one-way ANOVA followed by the Welch and Brown-Forsythe ANOVA test for multiple comparisons (GraphPad, Prism). ns, not significant; *p< 0.05; **p < 0.01; ***p < 0.001; ****p < 0.0001. (B) Immunoblot analysis of NF-ϰB and actin levels produced by the HDFs. Molecular weights are shown in kilodaltons (kDa). Representative data of three biological replicates are shown. Quantification of the NF-ϰB protein levels in each sample normalized to actin and relative to the NF-ϰB/actin signal intensity of HDFs alone treated with the negative control inhibitor (NC inh). Data represent the average of biological triplicates ± standard deviation. (C) Quantification of the picograms per milliliter (pg/mL) of IL6 secreted by the HDFs. Data represent the average of biological triplicates ± standard deviation. Statistical significance was determined by unpaired t test (GraphPad, Prism). ****p < 0.0001, (D) Immunoblot analysis of STAT1 and actin levels produced by the HDFs. Molecular weights are shown in kilodaltons (kDa). Representative data of four biological replicates are shown. Quantification of the STAT1 protein levels in each sample normalized to actin and relative to the STAT1/actin signal intensity of HDFs alone treated with the negative control mimic (NC mimic). Data represent the average of four biological replicates ± standard deviation. (E) Immunoblot analysis of TRAF6 and actin levels produced by the HDFs. Molecular weights are shown in kilodaltons (kDa). Representative data of three biological replicates are shown. Quantification of the TRAF6 protein levels in each sample normalized to actin and relative to the TRAF6/actin signal intensity of HDFs alone treated with the negative control inhibitor (NC inh). Data represent the average of biological triplicates ± standard deviation.

miR146a-5p was predicted to be an important negative regulator of the immune response to early *B*. *burgdorferi* infection. Our data demonstrated that miR146a-5p expression, as well as the expression of its mRNA targets, were upregulated in HDF cells in the presence of *B*. *burgdorferi*. To interrogate the putative regulatory role of miR146a-5p, the levels of miR146a-5p in HDF cells were manipulated by overexpression or inhibition. Transfection of HDF cells with a miR146a-5p mimic, resulted in overexpression of the miRNA up to a log2 FC of 9.3 in the control fibroblasts, and to a log2 FC of 6.3 in the fibroblasts stimulated with *B*. *burgdorferi* for 24 hours, both at an adjusted P value of <0.0001 (Fig 4A). Moreover, overexpression of miR146a-5p in the presence of *B*. *burgdorferi* resulted in a significant decrease in expression of the miR146a-5p target gene *IL6* (Fig 4B). Interestingly, expression of the gene encoding the proinflammatory chemokine CXCL10 in HDFs stimulated with *B*. *burgdorferi* for 24 hours was also significantly reduced with miR146a-5p overexpression (Fig 4B). While not statistically significant, cells treated with miR146a-5p mimic and *B*. *burgdorferi* showed a trend in the reduction of the expression levels of *NFϰB1*, *STAT1* and *TLR2* (Fig 4B). Although overexpression of miR146a-5p did not significantly affect NF-ϰB protein levels (Fig 4C), this treatment resulted in reduced levels of IL6 protein secreted by HDFs stimulated with *B*. *burgdorferi* (Fig 4D) and a trend in reduction of the levels of STAT1 protein in both the HDFs alone and HDFs stimulated with *B*. *burgdorferi* (Fig 4E). Conversely, the inhibition of miR146a-5p resulted in a significant increase in the level of *IL6* gene expression in both HDFs alone and HDFs stimulated with *B*. *burgdorferi* for 24 hours, compared to the cells treated with the negative control inhibitor (Fig 5A). No statistically significant changes in *STAT1*, *TLR2*, *NFϰB1* or *CXCL10* gene expression were observed for HDF cells transfected with the miR146a-5p specific inhibitor and stimulated with *B*. *burgdorferi*, although a trend of increased expression was observed for all four genes (Fig 5A). At the protein level, inhibition of miR146a-5p had no detectable effect on NF-ϰB (Fig 5B), however, the amount of secreted IL6 was significantly increased in fibroblasts transfected with the miR146a-5p inhibitor and stimulated with *B*. *burgdorferi* compared to *B*. *burgdorferi* stimulated HDFs treated with the negative control inhibitor (Fig 5C). Inhibition of miR146a-5p resulted in a significant increase in STAT1 protein levels in HDF cells alone but no change in HDF cells stimulated with *B*. *burgdorferi* (Fig 5D).

To explore the effect of miR146a-5p manipulation on the negative feedback regulation of NF-ϰB in the context of *B*. *burgdorferi* stimulation, the gene expression levels of *IRAK1*, *IRAK2* and *TRAF6* were analyzed. As expected, levels of *IRAK1* expression in HDF cells transfected with miR146a-5p mimic or miR146a-5p inhibitor demonstrated a significant decrease or increase, respectively, compared to the negative controls (Figs 4B and 5A). Yet, no *B*. *burgdorferi*-dependent change in *IRAK1* expression was detected. Although not statistically significant, trends of decreased expression levels of *TRAF6* in miR146a-6p mimic-treated and 24-hour *B*. *burgdorferi*-stimulated HDFs were observed (Fig 4B). In this model *TRAF6* expression was not significantly affected by inhibition of miR146a-5p (Fig 5A). The expression levels of *IRAK2* were unaffected by miR146a-5p overexpression (Fig 4B) but significantly increased following inhibition of miR146a-5p (Fig 5A). Consistent with our previous results, increased expression of *IRAK2* was also mediated by the presence of *B*. *burgdorferi*. The negative feedback regulation of miR146a-5p on TRAF6 is known to occur primarily at the posttranscriptional level [52, 53]. Therefore, we examined the effects of miR146a-5p manipulation on TRAF6 protein levels in HDF cells with and without *B*. *burgdorferi* stimulation for 24 hours. Similar to *TRAF6* gene expression levels, overall TRAF6 protein levels in this model were found to be low. No significant changes in TRAF6 protein were detected in the miR146a-5p mimic- or miR146a-5p inhibitor-treated samples, although a trend in decreased TRAF6 protein levels was observed with overexpression of miR146a-5p (Figs 4F and 5E).

## Discussion

MicroRNAs have emerged as important regulators of inflammation and are believed to play key roles in fine-tuning the level and kinetics of immune responses [54]. Lyme disease is a multistage inflammatory disease that initiates at the skin site of *B*. *burgdorferi* infection. The contribution of miRNAs to early *B*. *burgdorferi* infection was explored herein. Using a reverse-engineered miRNA target analysis approach and the published transcriptomes from erythema migrans skin lesions collected from early Lyme disease patients [22] or human dermal fibroblasts (HDFs) stimulated with *B*. *burgdorferi* [23], miR146a-5p was predicted to contribute to the regulation of the early host response to *B*. *burgdorferi*.

miR146a-5p is one of the most well characterized miRNAs for its role in negative regulation of the inflammatory response. It is well established that miR146a-5p responds to activation of Toll-like receptors (TLRs) by implementing a negative feedback loop via targeting of TRAF6, IRAK1, and IRAK2 [52, 55, 56]. This negative feedback loop reduces the level of TLR signaling and limits NF-κB function and its consequent transcriptional activation of proinflammatory signals such as IL8 and INFγ [57], thereby modulating severe inflammation after activation of the innate immune response [58]. Herein we established that stimulation of human dermal fibroblasts with *B*. *burgdorferi* resulted in increased expression of miR146a-5p. This finding was contrary to our prediction of *B*. *burgdorferi*-dependent repression of miR146a-5p based on the *B*. *burgdorferi*-dependent increased expression of the mRNA target genes in the transcriptome data sets. However, manipulation of miR146a-5p levels by overexpression or inhibition resulted in significant decrease or increase, respectively, in the levels of mRNA target gene expression, including *IL6*, *CXCL10* and *IRAK2*. Similar trends were observed for *NFϰB1*, *STAT1* and *TLR2*, together suggesting that miR146a-5p expression in *B*. *burgdorferi*-stimulated HDFs results in suppression of mRNA target gene expression. Kinetic analysis of *B*. *burgdorferi* inflammatory gene expression and induction of miR146a-5p revealed early induction of *NFϰB1*, *ICAM1*, *IL6* and *TLR2* followed by increased expression of miR146a-5p and *STAT1* as well as accumulation of IL6 and STAT1 protein. *B*. *burgdorferi* stimulation did not result in a detectable increase in the levels of NF-ϰB protein, consistent with the well-established post-translational mechanisms of regulation of NF-ϰB at the level of activity [59]. Overall, these findings are consistent with previous studies that reported similar changes in the inflammatory transcriptional response to *B*. *burgdorferi in vitro*, *in vivo* and *ex vivo*, characterized by a TLR and NF-ϰB inflammatory response [22, 23, 60–62].

The correlation of the transcriptional changes in inflammatory gene expression with a subsequent significant and gradual increase in the expression levels of miR146a-5p exhibited in our model is consistent with miR146a-5p being a known target of NF-ϰB regulation [40]. Investigation of the negative feedback, anti-inflammatory modulatory effect of *B*. *burgdorferi-*induced miR146a-5p on TLR-mediated signaling and the NF-ϰB pathway demonstrated that *IRAK2* expression was significantly increased in *B*. *burgdorferi*-stimulated HDFs and increased further still with miR146a-5p inhibition. In contrast, *IRAK1* expression was sensitive to miR146a-5p inhibition but unaffected by the presence of *B*. *burgdorferi*. *TRAF6* expression levels were low yet demonstrated significant miR146a-5p-mediated repression as well as a trend in *B*. *burgdorferi*-dependent repression. TRAF6 protein, the canonical target of miR146a-5p inhibition, demonstrated low levels in HDF cells and a trend in miR146a-5p- and *B*. *burgdorferi*-dependent reduction. Interestingly, a recent *in vitro* study of neuroborreliosis, using human brain microvascular endothelial cells challenged with *Borrelia bavariensis*, also showed an upregulation of *IRAK2* and *MYD88*, while no significant changes were detected for *IRAK1* or *TRAF6* expression [63].

The pattern of *B*. *burgdorferi*-mediated and miR146a-5p-mediated expression of *IRAK2* in our model paralleled that of *IL6*, *STAT1*, *TLR2* and *NFϰB1*. These data raise the possibility of a role for IRAK2 in the miR146a-5p-mediated negative feedback loop in HDFs stimulated with *B*. *burgdorferi*. IRAK2 has been primarily demonstrated to be a target of miR146a-5p in the context of viral infection leading to inhibition of RIG-1 mediated type I interferon production [38, 64, 65]. It is well established that *B*. *burgdorferi* induces type I interferon responses and these responses are correlated with dissemination ability and disease severity of various isolates [61, 66–70]. Although *IFNI* expression levels were below the level of detection in our study, IFN-responsive *STAT1* demonstrated *B*. *burgdorferi*-dependent induction in HDFs. However, experimental manipulation of miR146a-5p did not provide significant support for a role for the miRNA in modulation of *STAT1* gene expression and STAT1 protein levels in this model.

The immune modulatory effect of miR146a-5p has been shown to play a role in control of NF-ϰB activation in a mouse model of Lyme arthritis using miR146a-5p^-/-^ mice, demonstrating the importance of this regulatory network for resolution of inflammatory arthritis during persistent *B*. *burgdorferi* infection [18]. In this study TRAF6 was implicated as the target of miR146a-5p negative feedback regulation of NF-ϰB in primary mouse bone marrow derived macrophages stimulated with *B*. *burgdorferi* [18]. While little change in *TRAF6* expression was detected in our study, HDFs stimulated with *B*. *burgdorferi* suggested a trend in repression of *TRAF6* expression. Furthermore, treatment of *B*. *burgdorferi*-stimulated HDF cells with miR146a-5p mimic led to a trend in repression of TRAF6 protein levels. Although these results, suggest the possibility of TRAF6 as the target of miR146a-5p in *B*. *burgdorferi*-stimulated HDF cells, further study is warranted. Moreover, the targets of miR146a-5p regulation are known to vary depending on cell type [71] perhaps contributing to the differing results between the two studies. While in the mouse model miR146a-5p is important for controlling joint inflammation [18], in humans the synovial tissue of patients with postinfectious Lyme arthritis are characterized by significant inflammation as well as demonstrate high levels of miR146a-5p [19], suggesting that expression levels of miR146a-5p in the context of *B*. *burgdorferi* infection and Lyme disease must be tightly controlled as inflammatory dysregulation can arise from either too little or too much miR146a-5p.

**Fig 6:**
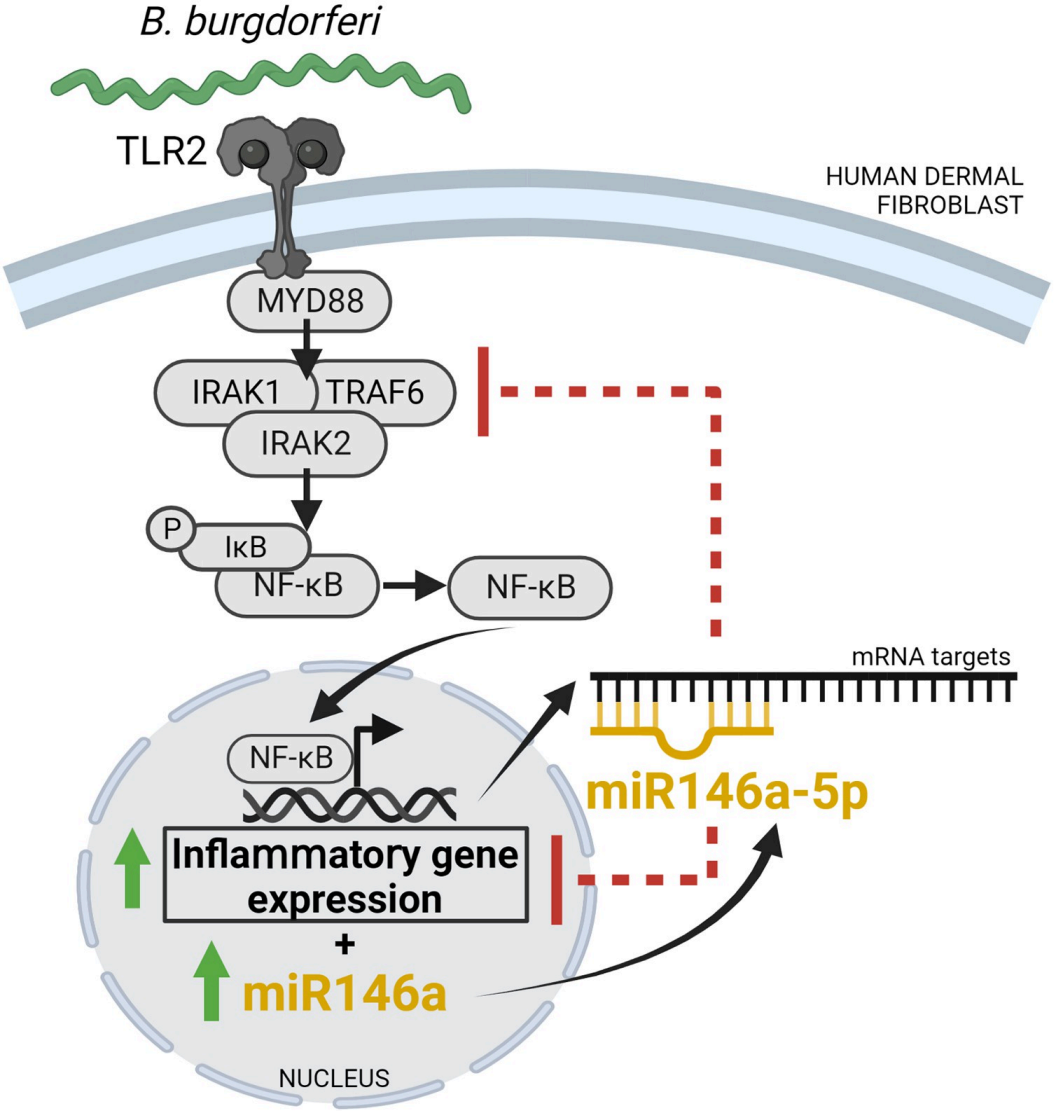
Model of miR146a-5p-mediated modulation of inflammatory gene expression in *B*. *burgdorferi*-stimulated human dermal fibroblasts. Stimulation of human dermal fibroblasts with *B*. *burgdorferi* results in increased expression of genes important for inflammation as well as the anti-inflammatory microRNA, miR146a-5p. Regulation by miR146a-5p reduces inflammatory gene expression levels perhaps by targeting mechanisms of NF-ϰB activation and/or the inflammatory gene transcripts directly.

In sum, we have shown that *B*. *burgdorferi* stimulation of human dermal fibroblasts results in increased expression of miR146a-5p, which in turn reduces the expression levels of genes important for inflammation perhaps by targeting mechanisms of NF-ϰB activation and/or the inflammatory gene transcripts directly (Fig 6). Together, these data suggest that miR146a-5p could have an important role in fine tuning the innate inflammatory response in the skin of humans infected by *B*. *burgdorferi*. Future studies aim to determine the consequences of miR146a-5p induction during the initial stages of infection for *B*. *burgdorferi* dissemination and Lyme disease pathogenesis.

## Supporting information

S1 Raw images Original immunoblot images for all of the immunoblot data presented in the manuscript. (PDF)

S1 Fig Expression levels of additional predicted miRNAs in HDFs stimulated with and without B. burgdorferi. (PDF)

S1 Table List of primer pairs used for mRNA qPCR. (PDF)

S2 Table List of TaqMan™ advanced miRNA assays, catalog number: A25576. (PDF)
