# Correction: *Borrelia burgdorferi*-mediated induction of miR146a-5p fine tunes the inflammatory response in human dermal fibroblasts

**DOI:** 10.1371/journal.pone.0322305

**Published:** 2025-04-08

**Authors:** Berta Victoria, Sarah A. Noureddine, Michael G. Shehat, Travis J. Jewett, Mollie W. Jewett

The Acknowledgements section of this article [1] is updated to

We thank the members of the M. Jewett and T. Jewett labs for critical feedback on this work. Fig 6 was created using BioRender.com. The authors received technical assistance with bioinformatics and the statistical analyses reflected in Tables 1, 2, 3 and Fig 1.
