# Supplementary material for: Borrelia burgdorferi-mediated induction of miR146a-5p fine tunes the inflammatory response in human dermal fibroblasts
Source: PLoS One. 2023 Jun 15;18(6):e0286959. doi: 10.1371/journal.pone.0286959 (PMC10270362; doi:10.1371/journal.pone.0286959)

Supporting Information
Original immunoblot images

***Borrelia burgdorferi*-mediated induction of miR146a-5p fine tunes the inflammatory response in human dermal fibroblasts.**

Berta Victoria[1], Sarah Noureddine[1], Michael G. Shehat[1,#a], Travis J. Jewett[1] and Mollie W. Jewett[1*]

[1]Division of Immunity and Pathogenesis, Burnett School of Biomedical Sciences, University of Central Florida College of Medicine, Orlando, Florida, United States of America.

[#a]Current Address: Department of Microbiology and Immunology, Faculty of Pharmacy, Alexandria University, Alexandria, Egypt.

*Address correspondence to Mollie W. Jewett, PhD, Mollie.Jewett@ucf.edu

Immunoblots were performed to determine the level of protein production of each target protein of interest normalized that of the actin control in each sample. All immunoblots were visualized using the LI-COR Odyssey scanner and software (Image Studio version 4). Immunoblot Figure 4C_ NFϰB Replicate 3 was stripped and reprobed for STAT1 (Figure 4E_STAT1 Replicate 3) and the actin signal from the NFϰB immunoblot used for the quantification analysis of the STAT1 signal. Similarly, immunoblots Figure 5B_NFϰB Replicate 2 and Figure 5B_ NFϰB Replicate 3 were stripped and reprobed for STAT1 (Figure 5D_STAT1 Replicate 3 and Figure 5D_ STAT1 Replicate 4) and the actin signal from the NFϰB immunoblots used for the quantification analysis of the STAT1 signal.

Figure 3B_NFκB_original blots

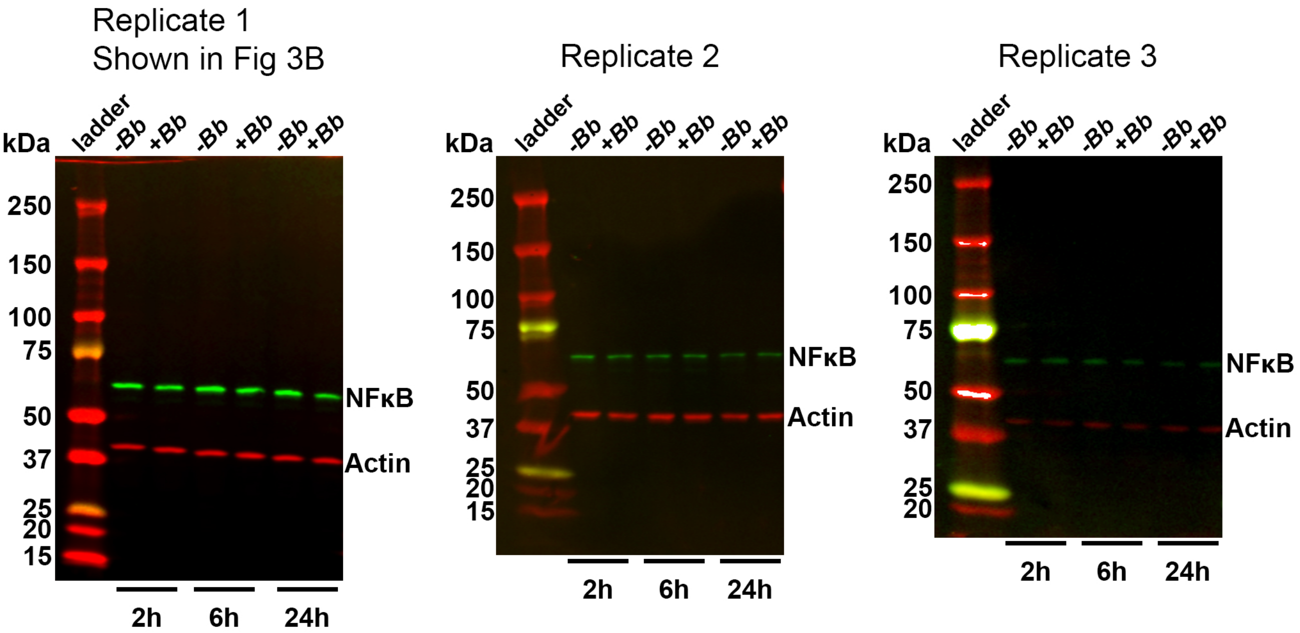

Figure 3D_STAT1_original blots

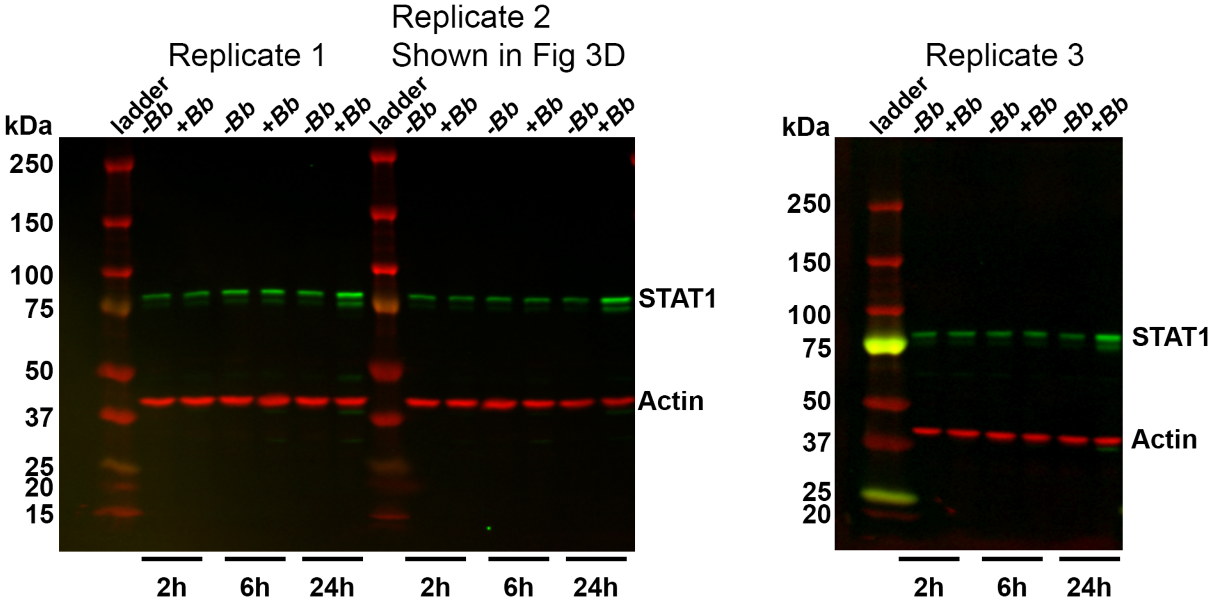

Figure 4C_NFκB_original blots

Replicate 1
Shown in Fig 4C

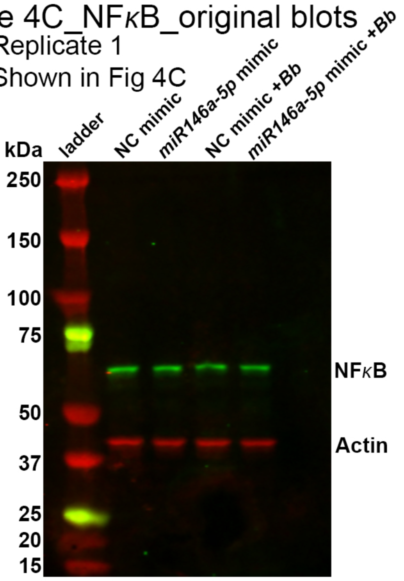

Replicate 2

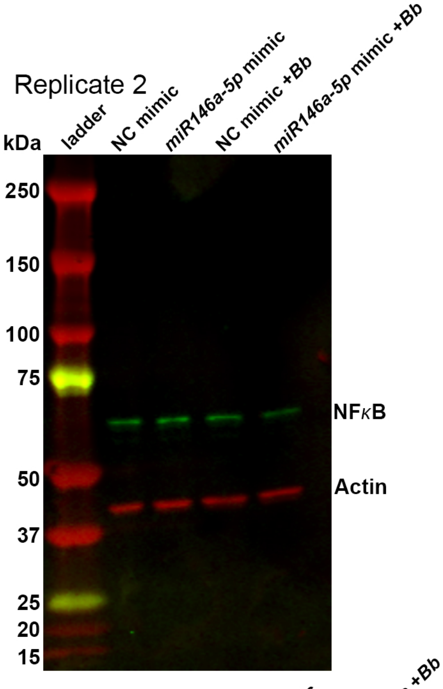

Replicate 3

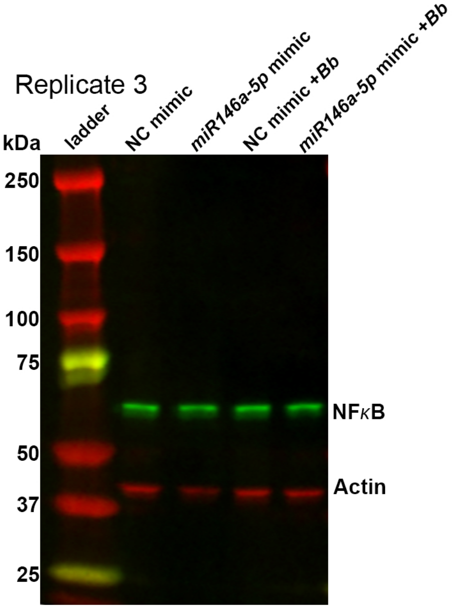

Figure 4E_STAT1_original blots

Replicate 1
Shown in Fig 4E

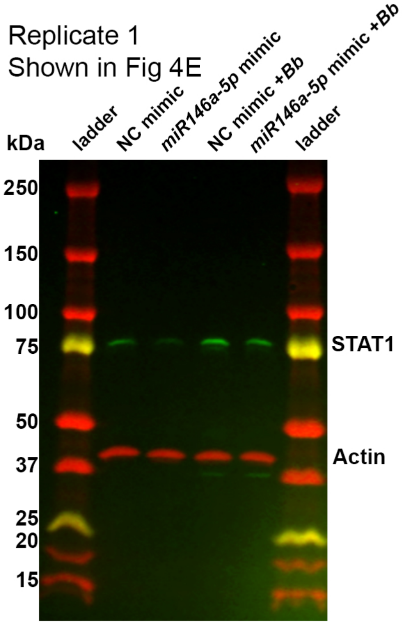

Replicate 2

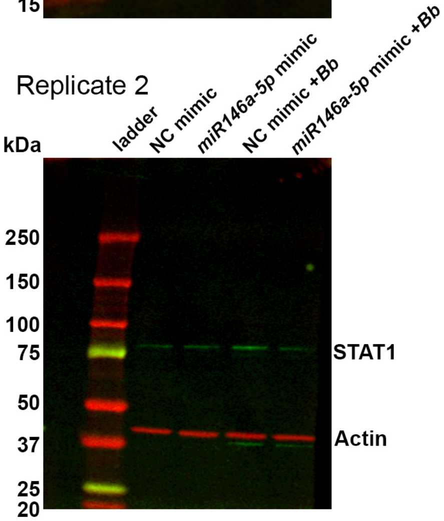

Replicate 3

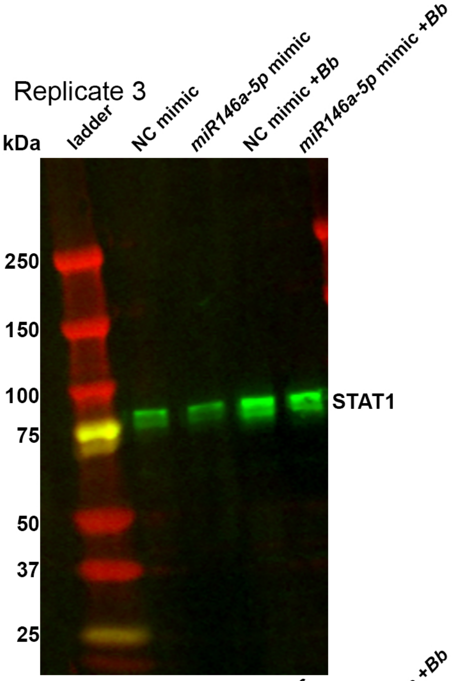

Figure 4F_TRAF6_original blots

Replicate 1

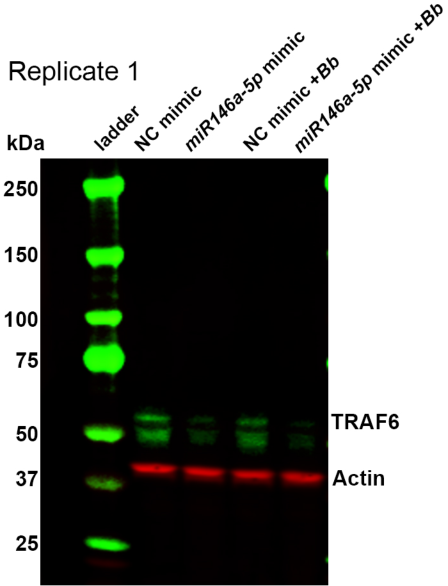

Replicate 2
Shown in Fig 4F

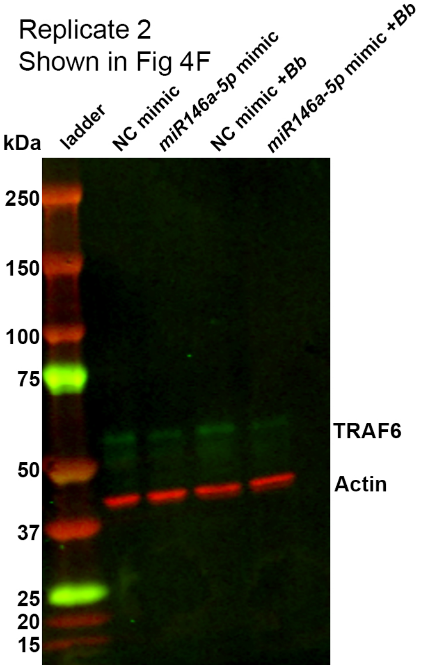

Replicate 3

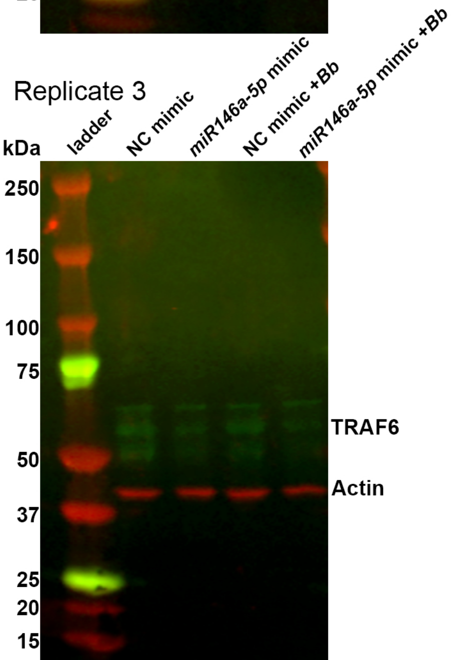

Figure 5B_NFκB_original blots

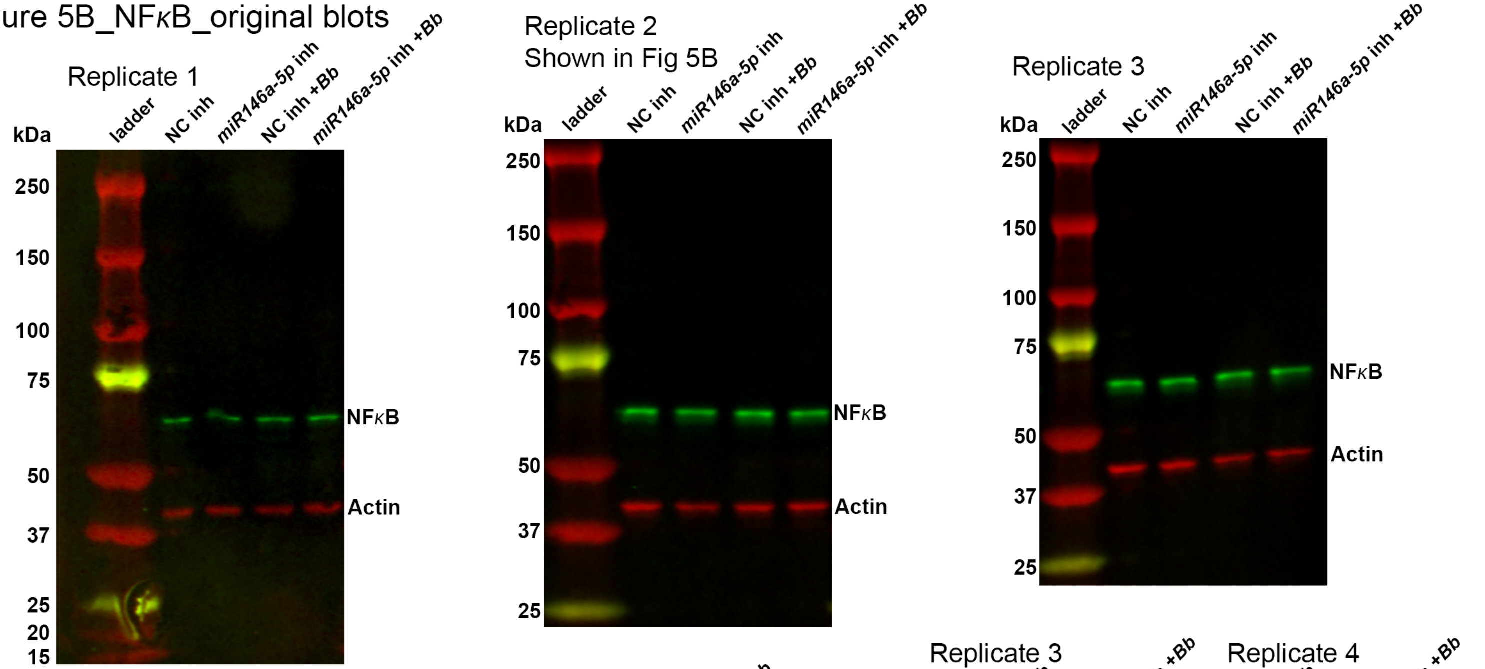

Figure 5D_STAT1_original blots

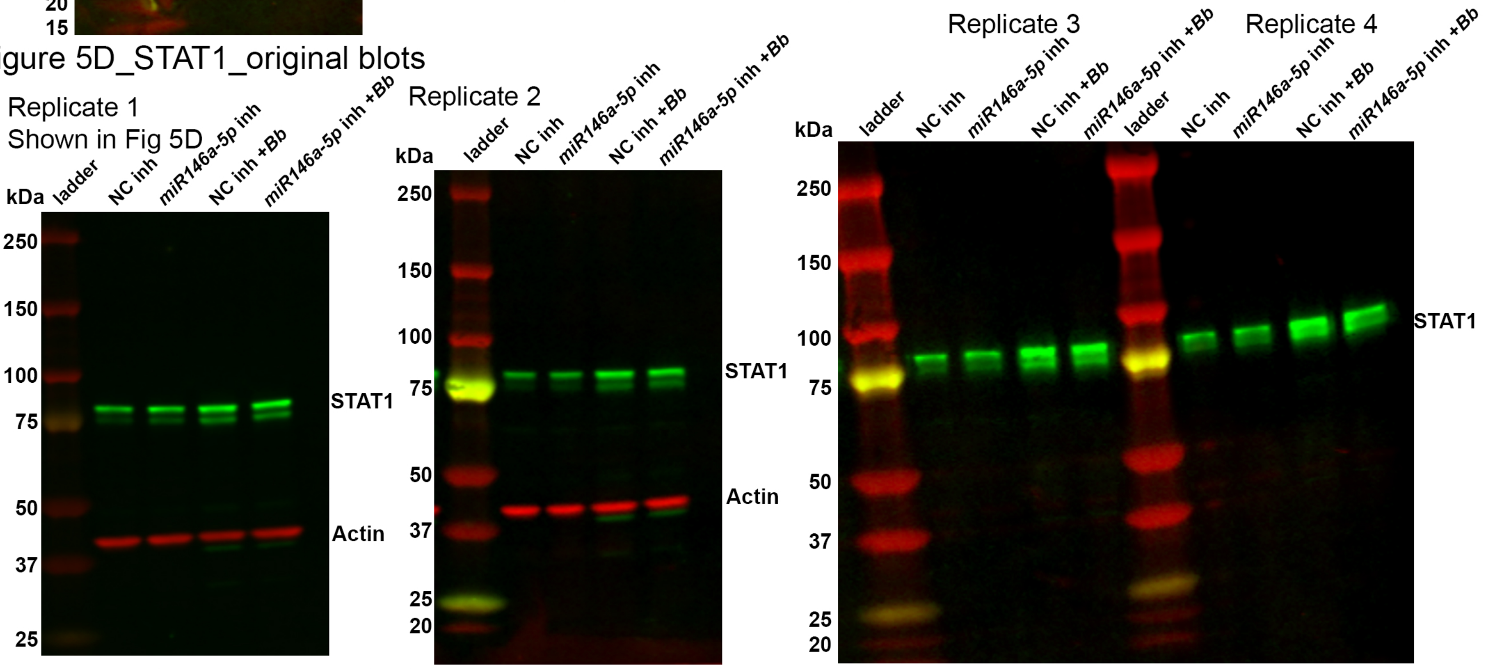

Figure 5E_TRAF6_original blots

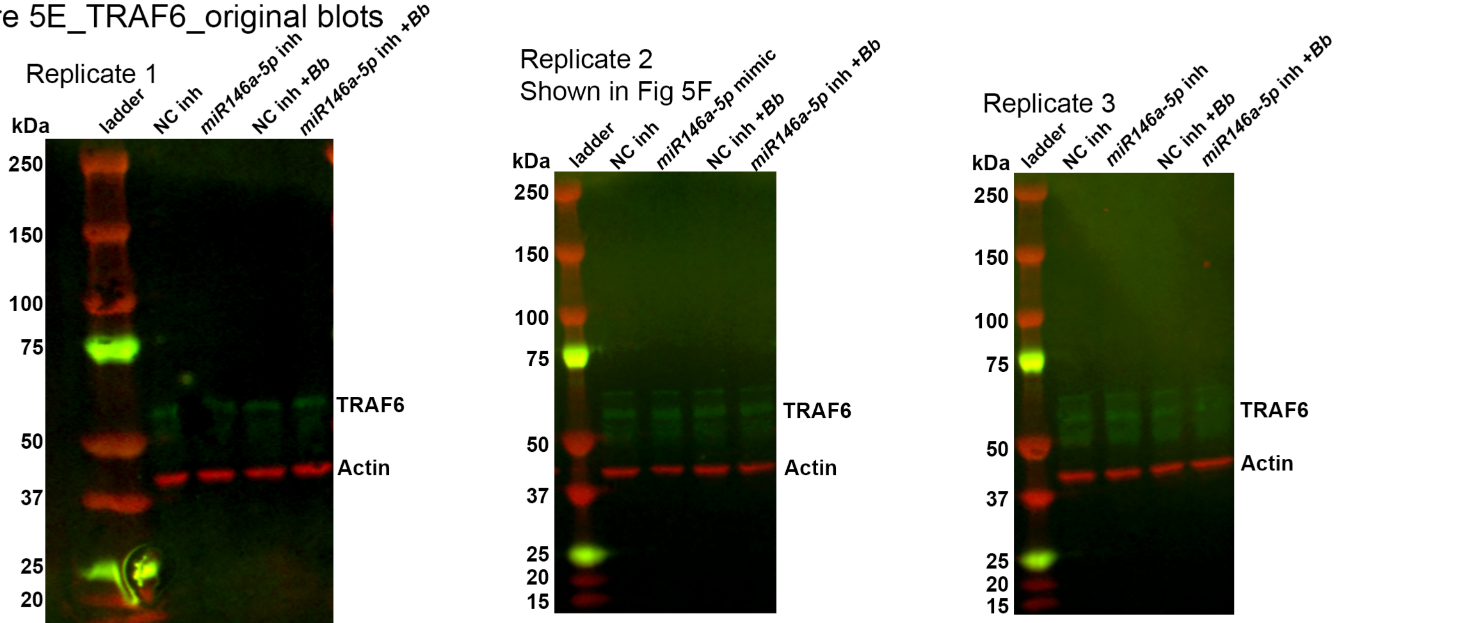

Supplement: S1 Raw images — (PDF) [file pone.0286959.s001.pdf]
