# Supplementary material for: Borrelia burgdorferi-mediated induction of miR146a-5p fine tunes the inflammatory response in human dermal fibroblasts
Source: PLoS One. 2023 Jun 15;18(6):e0286959. doi: 10.1371/journal.pone.0286959 (PMC10270362; doi:10.1371/journal.pone.0286959)
Supplement: S1 Fig — (PDF) [file pone.0286959.s002.pdf]

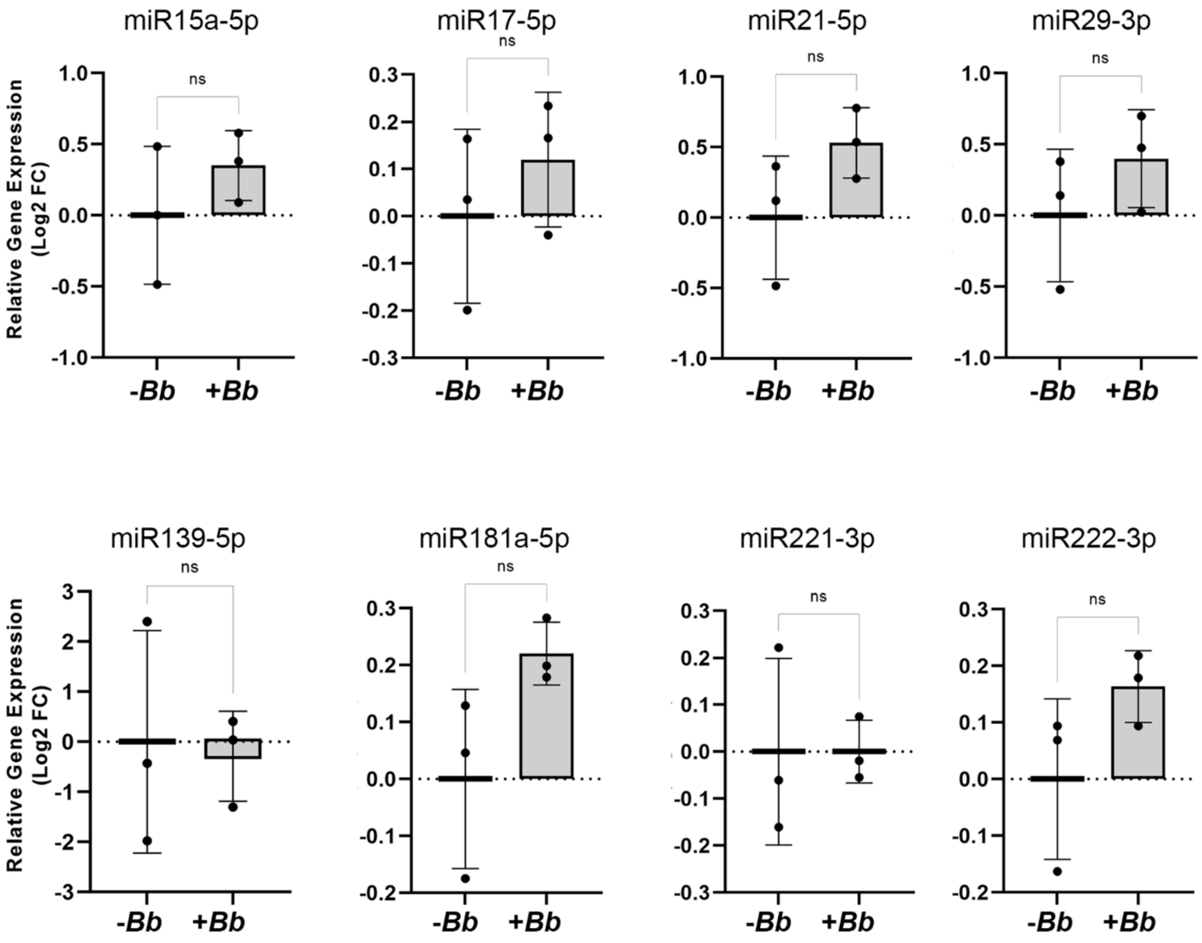

S1 Fig. Expression levels of additional predicted miRNAs in HDFs stimulated with and without *B. burgdorferi*. Data are presented as the relative log fold change in miRNA levels in HDFs co-incubated with *B. burgdorferi* (+*Bb*) compared with HDFs alone (-*Bb*). Data represent the average of biological triplicates ± standard deviation. Statistical significance was determined by unpaired t test (GraphPad, Prism). ns, not significant.
