# Supplementary material for: Borrelia burgdorferi-mediated induction of miR146a-5p fine tunes the inflammatory response in human dermal fibroblasts
Source: PLoS One. 2023 Jun 15;18(6):e0286959. doi: 10.1371/journal.pone.0286959 (PMC10270362; doi:10.1371/journal.pone.0286959)
Supplement: S1 Table — (PDF) [file pone.0286959.s003.pdf]

S1 Table. List of primer pairs used for mRNA qPCR.

| Gene ID | Primer sequence (5′-3′) | |
|---|---|---|
| | **Forward** | **Reverse** |
| STAT1 | CCATCCTTTGGTACAACATGC | AAAGCTGAGCCCATCGTG |
| IL6 | GCCCAGCTATGAACTCCTTCT | GCGGCTACATCTTTGGAATC |
| TLR2 | TCTCCCATTTCCGTCTTTTT | GGTCTTGGTGTTCATTATCTTC |
| ICAM1 | GAAGTGGTGGGGGAGACATA | CCCAATAGGCAGCAAGTTTC |
| CXCL10 | AAGCAGTTAGCAAGGAAAGGTC | GTCCATCCTTGGAAGCACTGCA |
| NFKB1 | CCTGCTCCTTCCAAAACACT | CGGTGTAGCCCATTTGTCTC |
| CCL2 (MCP-1) | CGGTGTAGCCCATTTGTCTC | CGGCAGAGCCTGAACTAGAA |
| TIMP3 | CATCCGCCTTTCCCTTTAG | GGTTTCTCTCCATCACTTCTGG |
| RECK | AATCCTTGCCCTGCCAATGAGC | GCACCTGGATTAGTGTCCCTTG |
| WEE1 | GAGCTTCCTGAGCCGACA | GCAGGGCGAGAAGATCAG |
| FOS | GGGGCAAGGTGGAACAGT | TCTCCGCTTGGAGTGTATCA |
| COL3A1 | CTGGTGGTAAAGGCGAAATG | AGTCCAGGAGCACCATTAGC |
| IRAK1 | ACATCAAGACGGGAAGGCTG | GTGCTCTGGGTGCTTCTCAA |
| TRAF6 | CCTTTGGCAAATGTCATCTGTG | CTCTGCATCTTTTCATGGCAAC |
| IRAK2 | AACTTGTGGACCTCCTGTGC | AGAGTCAGGGAAGGCTGGAA |
| MYD88 | GCTCATCGAAAAGAGGTGCC | ACTTGATGGGGATCAGTCGC |
| IL-8 | ACTCCAAACCTTTCCACCCC | TTCTCAGCCCTCTTCAAAAACT |
| GAPDH | AGCCACATCGCTCAGACAC | GCCCAATACGACCAAATCC |
| RPL2 (RNApol2) | GCACCACGTCCAATGACAT | GTGCGGCTGCTTCCATAA |
