# Supplementary material for: Borrelia burgdorferi-mediated induction of miR146a-5p fine tunes the inflammatory response in human dermal fibroblasts
Source: PLoS One. 2023 Jun 15;18(6):e0286959. doi: 10.1371/journal.pone.0286959 (PMC10270362; doi:10.1371/journal.pone.0286959)
Supplement: S2 Table — (PDF) [file pone.0286959.s004.pdf]

S2 Table. List of TaqMan™ Advanced miRNA Assays, catalog number: A25576.

miRBase ID	Assay ID
hsa-miR-146a-5p	478399_mir
hsa-miR-222-3p	477982_mir
hsa-miR-15a-5p	477858_mir
hsa-miR-17-5p	478447_mir
hsa-miR-221-3p	477981_mir
hsa-miR-21-5p	477975_mir
hsa-miR-29b-3p	478369_mir
hsa-miR-139-5p	478312_mir
has-miR-181a-5p	477857_mir
hsa-miR-155-5p	483064_mir
hsa-miR-186-5p	477940_mir
